# Quantification of acceleration as activity counts in ActiGraph wearable

**DOI:** 10.1038/s41598-022-16003-x

**Published:** 2022-07-13

**Authors:** Ali Neishabouri, Joe Nguyen, John Samuelsson, Tyler Guthrie, Matt Biggs, Jeremy Wyatt, Doug Cross, Marta Karas, Jairo H. Migueles, Sheraz Khan, Christine C. Guo

**Affiliations:** 1 ActiGraph LLC, 49 East Chase St., Pensacola, FL 32502 US; 2 Department of Biostatistics, Harvard T.H. Chan School of Public Health, Harvard University, 677 Huntington Avenue, Boston, MA02115 US; 3 Department of Biosciences and Nutrition, Karolinska Institute, Huddinge, Sweden; 4 Department of Physical Education and Sports, Faculty of Sport Sciences, PROFITH “PROmoting FITness and Health through physical activity” Research Group, Sport and Health University Research Institute (iMUDS), University of Granada, Granada, Spain; 5 Athinoula A. Martinos Center for Biomedical Imaging, MGH/MIT/Harvard, 149, 13th St., Boston, MA 02129 USA

**Keywords:** Computational platforms and environments, Data integration, Data processing, Software

## Abstract

Digital clinical measures based on data collected by wearable devices have seen rapid growth in both clinical trials and healthcare. The widely-used measures based on wearables are epoch-based physical activity counts using accelerometer data. Even though activity counts have been the backbone of thousands of clinical and epidemiological studies, there are large variations of the algorithms that compute counts and their associated parameters—many of which have often been kept proprietary by device providers. This lack of transparency has hindered comparability between studies using different devices and limited their broader clinical applicability. ActiGraph devices have been the most-used wearable accelerometer devices for over two decades. Recognizing the importance of data transparency, interpretability and interoperability to both research and clinical use, we here describe the detailed counts algorithms of five generations of ActiGraph devices going back to the first AM7164 model, and publish the current counts algorithm in ActiGraph’s ActiLife and CentrePoint software as a standalone Python package for research use. We believe that this material will provide a useful resource for the research community, accelerate digital health science and facilitate clinical applications of wearable accelerometry.

## Introduction

The rapid advances in computing and micro-electromechanical systems (MEMS) technology have created new opportunities for monitoring health in people’s free-living environment over extended time periods. In particular, as wearable devices are capable of quantifying patients’ behaviors continuously with minimal burden, they can provide more comprehensive, ecological and objective health information than what is possible with conventional in-clinic and questionnaire-based assessments^1–3^. For these reasons, the use of wearable devices for clinical purposes has seen rapid expansion in recent years and is expected to grow further in the upcoming years^4^. One of the most used outcomes provided by wearable devices is accelerometry-based physical activity measures. Physical activity measures have been used in clinical trials and shown superiority in detecting treatment related changes over conventional endpoints^5^. Progress has also been made in the qualification of digital endpoints based on counts of physical activity with both US and EU in respiratory and cardiovascular diseases^6,7^.

While the origin of the term “count” is not documented, it likely goes back to the modification and use of watches for measuring activity, specifically hyperactivity in children where pedometers were too large^8–11^. This was achieved by a winding weight that was free to pivot about its axis and therefore sensitive to acceleration while being connected to the hands of the watch via gears, causing the minutes and hours hands to tick when the watch experienced acceleration and thus allowing for reading the accumulated “counts” of movements from the watch the same way as one would read the time^11^. As electronics technology evolved, solid-state analog-to-digital converters became widely available and allowed for enhanced acceleration measurements and storing the activity data in a digital format inside the watch^12–14^. The first activity watch provided by ActiGraph was model AM7164 (ActiGraph, Pensacola (FL), USA) that used a uniaxial piezoelectrical accelerometer in the form of a mechanical lever^14^. To remove artifacts unrelated to human movement, subsecond-level measurements of acceleration (expressed in *g*) were passed through an analog band-pass filter before it was sampled at 10 Hz and quantized by an 8-bit analog-to-digital converter, thus yielding 256 distinct levels of acceleration. Each level beyond 128 is considered to be 1 count. These were then summed within given time intervals, or “epochs”, and the resulting counts were stored on the device (hence, each epoch could contribute up to $128 \times 10 \times \textit{the duration of the epoch}$ counts to the total).

The counts unit is thus a measure that quantifies acceleration within a time interval, or “epoch”, with one epoch typically being 10–60 s long. The reliance on epoch-based counts was a necessity in earlier models, due to the limitation of on-board storage and battery capacity. While this limitation was overcome with hardware improvements and ActiGraph has been able to provide multi-day raw data since 2010, digital clinical measures and validation evidence continue to evolve around counts. Because the algorithms that transform the raw accelerometer data into counts vary across devices, many of which have also been held proprietary, digital measures based on counts are device dependent, making it difficult to compare results across clinical studies and establish reproducibility and validation evidence^15–17^. The obscurity of the count algorithms has also led to the common misconception that “counts” is a universal unit of measurement that is the same across devices, where even with an algorithm such as the one described below, variations in parameters such as the length of each epoch or the precision of the quantization process can lead to vastly different counts. Therefore, the advances of digital health science could be greatly facilitated with a higher level of algorithm transparency which would allow it to be used across multiple devices.

ActiGraph devices (ActiGraph, Pensacola, FL, USA) have been the most-used wearable accelerometry, or actigraphy, devices for over two decades, with more than 20,000 papers published using ActiGraph devices by the end of 2021. While the original methods for computing counts in the earlier models have been published^14^, the detailed algorithms and their evolution over the years have not been made public. Several studies have attempted to reverse engineer the ActiGraph counts algorithms or relate them to the counts algorithms of other devices^16–19^. While these studies have been critically important to gain further understanding of acceleration counts and how it relates across devices, they have been limited by the lack of access to the ActiGraph firmware and software code and device documentation. In this article, we address this gap by presenting an overview of the counts algorithms of ActiGraph devices along with a detailed description of the counts algorithm in both the ActiLife software (ActiGraph, Pensacola, FL, USA) and the CentrePoint cloud service (ActiGraph, Pensacola, FL, USA). An open-source Python package is also made available for use by the research community. By doing so, we hope to facilitate reproducibility efforts and enhance transparency in the field of wearable accelerometry and accelerate its clinical use.

## Results

**Figure 1:**
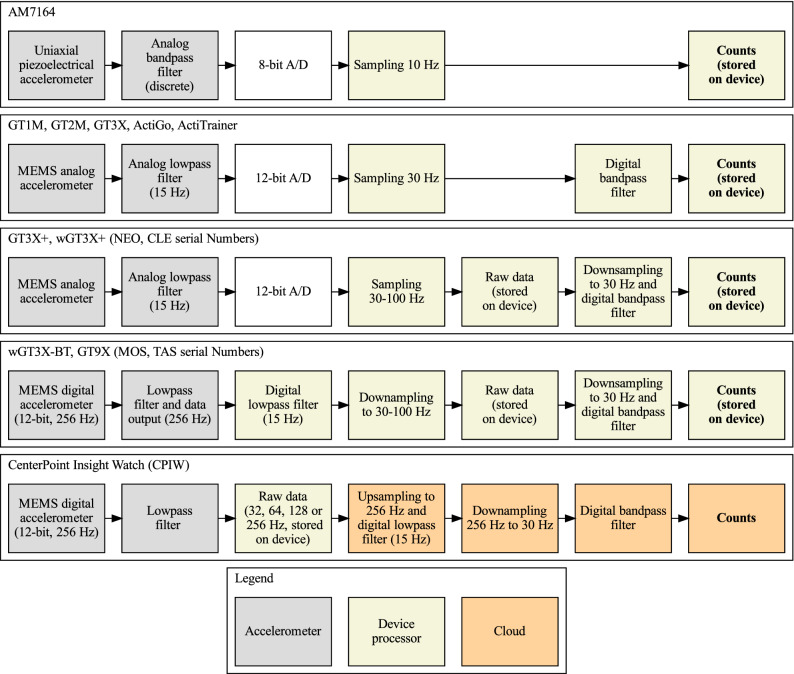
Conceptual schematic of the computational pipeline for generating counts for five generations of ActiGraph accelerometer models (rows). Raw data measures acceleration in free fall acceleration (g) units. Functions performed by the microprocessor, the accelerometer and in the CentrePoint cloud are highlighted in yellow, gray and orange, respectively. The analog band-pass filters in AM7146 are implemented using a series of cascaded op amp circuits, the half magnitude points for the lower and upper cutoff frequencies are 0.21 and 2.28 Hz, respectively^14^. For the analog low-pass filters the − 3 dB cutoff frequency is shown in parenthesis for each block. A/D is the analog-to-digital converter (white blocks), shown together with the bit size of the quantizer. The counts data blocks are highlighted in boldface.

**Figure 2:**
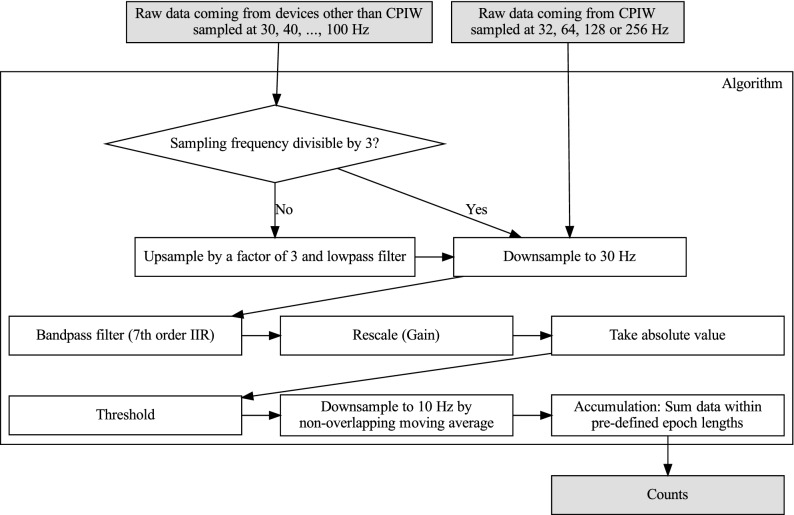
Flowchart showing the process of converting raw data into counts.

We first present here a flowchart overview of the processing pipelines to derive counts across five generations of ActiGraph models (Fig. 1). It is important to note that all counts algorithms outlined here produce counts after band pass filtering the raw signal around frequencies compatible with human activity. The analog band-pass filter’s maximum gain is at 0.759 Hz, and goes down to − 6 dB at 0.212 Hz at 2.148 Hz. The digital version of this filter (in devices other than AM7164) replicates the analog version^14^. The conversion of the raw data into counts depends therefore not only on the amplitude of the raw data but also on its spectral content. In the CPIW model (bottom row in Fig. 1), the accelerometer applies an anti-aliasing low-pass filter before the signal is sampled to respect Nyquist’s theorem^20^, and both the low-pass − 3 dB cutoff frequency and the sampling frequency are adjustable; the possible output data rates are 32, 64, 128, and 256 Hz and the associated low-pass cutoff frequencies are 16, 16, 32, and 64 Hz, respectively. The process of converting raw data to counts (highlighted in bold in Fig. 1) for the newer models wGT3X-BT, GT9X, and CPIW are further elucidated below (Fig. 2).

1. Only for CPIW; resample data to 30 Hz (from 32, 64, 128 or 256 Hz). Otherwise assert that sampling frequency is between 30 and 100 Hz and a multiple of 10 Hz.
2. If sampling frequency is not a multiple of 30, then up-sample by a factor of 3 to make it a multiple of 30 and low pass filter. This is achieved by the following steps:
  $$\begin{aligned} v&= \text {array of size } (3 \times \text {length of raw signal}) \text { filled with } 0 \\ w&= v\\ v_{3\times i}&= \text {raw signal}_i ~\forall i\in \left[ 0, \ldots , \text {size}(\text {raw signal})\right] \\ w_i&= \frac{3\pi }{\pi +3\times 2} v_i+\frac{3\pi }{\pi +3\times 2}v_{i-1} +\frac{2\times 3-\pi }{\pi +2\times 3}w_{i-1} \forall i. \end{aligned}$$
3. Down-sample to 30 Hz. Since the signal is a multiple of 30 Hz, the down-sampling is straightforward by keeping every $m\text {th}$ sample, where m is the up-sampled frequency divided by 30;
  $$\begin{aligned} m&= \frac{\text {up-sampled frequency}}{30} \\ x_i&= w_{m\times i}~\forall i. \end{aligned}$$
4. Band-pass filter the down-sampled signal. This is done by firstly defining the filter (a 7th order IIR filter), which can be described by the rational transfer function in the z-transform domain as:
  $$\begin{aligned} \hat{X}(z)=\frac{\sum _{i=0}^{7}b_iz^{-i}}{\sum _{i=0}^{7}a_iz^{-i}}X(z), \end{aligned}$$
  where $a_0=1$, $a_1=-\,3.63$, $a_2=5.04$, $a_3=- \,3.10$, $a_4=0.506$, $a_5=0.324$, $a_6=- \,0.157$, $a_7=0.0195$, and $b_0=-\, 0.00934$, $b_1=- \,0.0255$, $b_2=- \,0.00424$, $b_3=0.0442$, $b_4=0.0365$, $b_5=-\, 0.0119$, $b_6=- \,0.0229$, $b_7=-\, 0.00679$^14^. These coefficients have been rounded to 3 significands—please refer to the public code for the exact numbers. The initial condition that generates a steady state to a step response of this filter is then found, and the down-sampled signal $x_i$ is filtered using this initial state and the filter described above, resulting in the filtered signal $\hat{x}_i$.
5. Rescale the filtered signal by a factor *a* ($a=\frac{3.0 / 4096.0}{2.6 / 256.0} \times 237.5 \approx 17.127404$), in order to replicate the range of the AM7164.
  $$\begin{aligned} \tilde{x}_i=a\hat{x}_i \end{aligned}$$
6. Rectify the rescaled signal:
  $$\begin{aligned} \bar{x}_i =|\tilde{x}_i|. \end{aligned}$$
7. Threshold the rectified signal so that all entries greater than 128 is set to 128 and all entries smaller than 4 are set to 0:
  $$\begin{aligned} \overrightarrow{x}_i = {\left\{ \begin{array}{ll} 128 &{} (\bar{x}_i>128) \\ \text {floor}(\bar{x}_i) &{} (4\le \bar{x}_i\le 128) \\ 0 &{} (\bar{x}_i<4). \end{array}\right.}. \end{aligned}$$
8. Further down-sample the signal and low-pass filter to 10 Hz by a non-overlapping moving average:
  $$\begin{aligned} \check{x}_j=\left\{ \text {floor}(\frac{\overrightarrow{x}_i+\overrightarrow{ x}_{i+1}+\overrightarrow{x}_{i+2}}{3})\right\} _{i=0,3,6,\ldots}. \end{aligned}$$
9. Finally, find the counts by summing the down-sampled signal within the predefined epoch length l (s) for each axis:
  $$\begin{aligned} \text {counts}_i=\sum _{j=i\cdot 10\cdot l}^{(i+1)\cdot 10\cdot l}\check{x}_j. \end{aligned}$$

**Figure 3:**
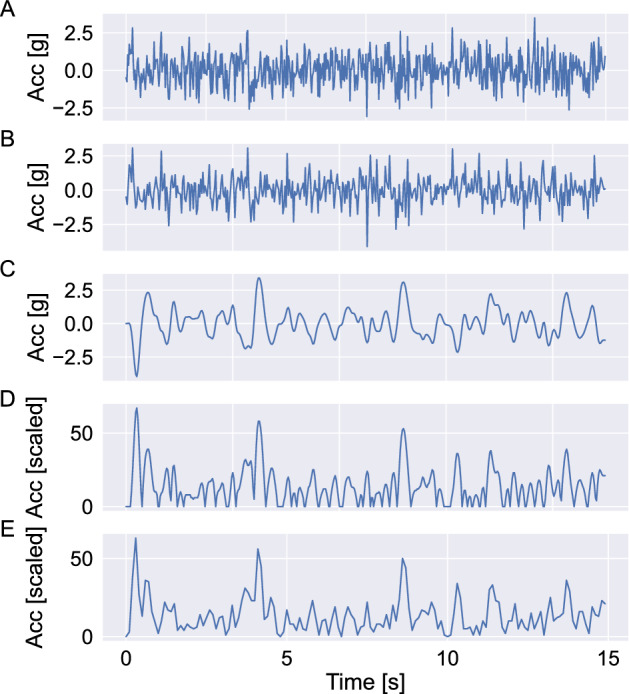
Example waveform and the resulting waveforms after each processing step. (**A**) The original waveform, at 40 Hz sampling rate. (**B**) The waveform down-sampled to 30 Hz. (**C**) The waveform after band-pass filtering. (**D**) The waveform scaled and rectified. (**E**) The waveform down-sampled to 10 Hz.

Figure 3 presents an example waveform along with the result of each processing step. The python code was validated to the ActiLife software and CentrePoint service using synthetic raw data as described in the methods section. Validation was performed on four cases with 1000 simulations each: corresponding to combinations of raw data at 40 or 30 Hz sampling rate and epoch of 10 or 30 sec duration. We did not find any differences between the values returned by any of the three implementations.

## Discussion

**Figure 4:**
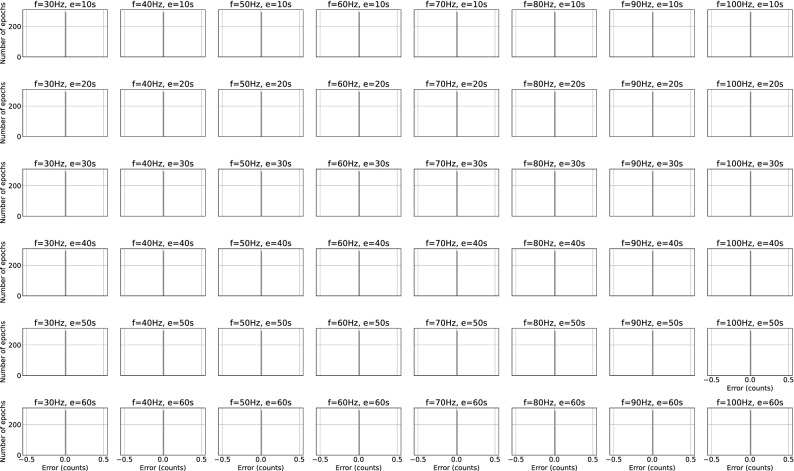
Comparison of counts generated by our python code and those generated by CenterPoint. Each plot corresponds to a combination of data sampling rate and epoch length. From top to bottom, epoch length goes from 10 to 60 s. From left to right, sampling frequency goes from 30 to 100 Hz. In all cases, the counts are identical between the two implementations.

Remote clinical assessments based on wearable devices have the potential to transform clinical development and care^4^. This potential requires robust validation evidence and can be facilitated with a greater level of algorithm transparency. Here we provided an overview of the counts algorithms of five generations of ActiGraph devices (Fig. 1), released a Python package that replicates the counts algorithm in ActiGraph’s ActiLife software under an open source license, validated the Python code to the output from ActiLife (Fig. 4) and detailed the numerical steps of arriving at counts in pseudocode (Fig. 2). We believe that this material will be an important and useful resource for the research community.

Several kinds of counts algorithms have been used in the literature: Time above threshold, zero-crossing and digital integration^15^. While the time above threshold and zero-crossing techniques have mostly been used in older devices, all ActiGraph models have used the digital integration technique, which is thought to be superior to the other counts techniques^12^ in terms of component size, power consumption, and accuracy in presence of noise. The processing steps of this technique differed between devices, as successive generations of ActiGraph devices made use of the latest advances in hardware and software technology. The computation processes have gradually evolved to being done in the CentrePoint cloud, and less so in the device microprocessor. Care has been taken in order to maintain comparability between counts extracted from different devices, so that the activity counts computed from the same acceleration input would stay consistent across generations of hardware and software platforms^21–23^ although, due to the vastly different technologies involved, differences have been found notably in minutes of vigorous activity per day^24^. This attention to data compatibility due to platform upgrades in clinical investigation has also been noted in the recent FDA draft guidelines on digital health technologies^25^.

Brłnd et al.^18^ tried to reverse engineer the ActiLife counts algorithm by reproducing the counts output from raw data input. The ActiGraph digital band-pass filter was approximated using Matlab’s *invfreqz* function. The reverse engineered counts algorithm was then applied to raw data from a different activity monitor; Axivity AX3 (Axivity, Newcastle UK). Fig. 1 in their paper^18^ outlines the steps in the derived counts algorithm which corresponds rather well with Fig. 2 in the present paper which details the ActiLife counts algorithm, although many of the computational steps happen in a different order. The ActiLife digital band-pass filter that was approximated in that paper^18^ is different from the one implemented in ActiLife; the ActiLife band-pass filter is a $7\text {th}$ order IIR filter while the one estimated in their study is a 20-order filter. They found generally high concordance between this output and the output from ActiLife applied to raw data from the GT3X+ monitor (not statistically significant from zero for mechanical validation experiment and a 2.2% mean difference from free-living conditions). However the 95% limits of agreement for the Brłnd counts vs the ActiLife counts ranged from [− 49, 41] counts/10s, to [− 156, 140] counts/10s across participants. The errors found between their approximation and the “true” counts also increased as the intensity of activity increased. Furthermore, there was a significant difference between their intensity classification and the one based on ActiGraph counts. In any case, even if we assume the Brłnd counts are comparable enough to the ActiLife counts to be used in studies with different monitors, those counts have not been widely applied in other studies and their clinical implications have not been investigated.

With the many versions of activity counts and additional open source activity metrics, such as Euclidean Norm Minus One (ENMO), Activity Index (AI)^26,27^, it is important to apply the cut points developed for the right type of activity counts. On the other hand, since there is generally high correlation between these activity metrics, it is feasible to compare the findings using different metrics with relatively simple conversion factors. In an experiment under free-living condition, Paul et al.^16^ showed that there was a significant difference in the counts output between the ActiGraph AM7164 monitor and the ones of Actical (MM; Mini-Mitter Co.) and it can be reduced significantly by the conversion equations they developed. Straker and Campbell^17^ also presented a linear conversion equation relating the vertical component of the ActiGraph GT3X model with the Actical counts.

To further facilitate the use and comparability of ActiGraph counts, we here also publish the codes in an open source Python package. The code validation showed perfect correspondence between ActiLife, CenterPoint, and the published Python code (see Fig. 4). Although we expect and indeed observe small differences between the intermediary results of different implementations due to the nature of working with floating point numbers, these differences had no effect on the final results of these algorithms.

Quantifying acceleration in ‘counts’ started due to technical limitations in the earliest wearable devices but has demonstrated its value for clinical research and is still omnipresent in the field of wearable accelerometry. Counts is not a universal unit, and the relationship between raw acceleration data and counts is complicated and varies between device models. Therefore, detailed understanding of the computation from raw accelerometer data to counts is necessary for interpreting clinical outcomes across studies and advancing the clinical application of wearable technology. In addition, proper demonstration and documentation of signal processing steps are necessary in obtaining regulatory agreement in the use of digital health technologies tools in clinical trials and/or medical devices. We hope that by publishing and detailing the counts algorithms of ActiGraph devices in this study, we could help accelerate digital health science and facilitate clinical applications of wearable accelerometry.

## Methods

Internal documentation of ActiGraph devices was reviewed to catalog the different processing steps to convert from raw data to counts. The code for converting raw data into counts in the ActiLife software was inspected along with the firmware documentation for ActiGraph models wGT3X-BT, GT9X, and CentrePoint Insight Watch (CPIW). The ActiLife counts algorithm was translated into a standalone Python package which converts raw data into counts at user-defined sampling frequencies and epoch lengths. The Python code was then converted into pseudocode, i.e. human-understandable basic instructions that can easily be translated back into any programming language. The code and pseudocode thus apply to wGT3X-BT, GT9X, and CPIW, although CPIW requires a down-sampling step before the computational pipeline described in the pseudocode can be applied, since the sampling rate is different in CPIW compared to the wGT3X-BT and GT9X models.

The Python code (Python 3.7.6) was validated to the counts algorithm in ActiLife (version 6.13.4) and in CentrePoint (version 3.29.0) by comparing counts output from the Python code with that of the ActiLife software and CentrePoint using the same raw input data, sampling frequency and epoch length. The raw input data were simulated Gaussian white noise and therefore consisted of a broad range of frequencies. The code was tested for all admissible sampling frequencies of these models (30, 40,…, 100 Hz) and varying epoch durations.

## Data Availability

The count algorithm and the code to reproduce and plot the validation data presented in Fig. 4 are publicly available at https://github.com/actigraph/agcounts. The code is also available as a python package. The parser for GT3x files is available at https://github.com/actigraph/pygt3x.
